# Editorial for the Special Issue “Natural Products for Antimicrobial and Drug Delivery: A Look into the Future”

**DOI:** 10.3390/pharmaceutics17070857

**Published:** 2025-06-30

**Authors:** Hélcio Silva dos Santos

**Affiliations:** Center for Exact Sciences and Technology, Vale do Acaraú University, Sobral 62040-370, Brazil; helcio_santos@uvanet.br

## 1. Introduction

The convergence of antimicrobial resistance and the ever-present need for targeted drug delivery is pushing researchers to explore nature’s vast collections of compounds with renewed interest. Natural products, from plant extracts to microbial metabolites, offer a tantalizing array of possibilities for tackling these critical challenges. Their inherent biocompatibility, structural diversity, and, in some cases, their long history of traditional use make them compelling candidates for both direct antimicrobial agents and innovative drug delivery systems [1]. The future of natural product-based antimicrobials lies not only in the rediscovery of forgotten remedies but also in the application of cutting-edge technologies. High-throughput screening, genomics-guided natural product discovery, and advanced analytical techniques are accelerating the identification of novel antimicrobial compounds with unique mechanisms of action [2]. The rise in multidrug-resistant bacteria necessitates a move beyond conventional antibiotics, and natural products offer the potential to circumvent existing resistance mechanisms or synergize with current therapies. Imagine nano-encapsulated plant extracts targeting biofilms or engineered bacteriophages carrying natural product-derived toxins—these are the kinds of innovative approaches being explored. Furthermore, natural products have proven invaluable in the development of advanced drug delivery systems [3]. Liposomes, nanoparticles, and microemulsions derived from natural sources offer enhanced biocompatibility, biodegradability, and the ability to target specific tissues or cells. For example, polysaccharides like chitosan and alginate are being used to create sustained-release formulations, while plant-derived lipids are being incorporated into liposomes to improve drug encapsulation and stability. The beauty of these systems lies in their ability to protect drugs from degradation, improve their bioavailability, and reduce off-target effects, ultimately enhancing therapeutic efficacy and patient outcomes [4,5,6]. Looking ahead, the successful translation of natural product-based antimicrobials and drug delivery systems will require a multidisciplinary approach. Collaboration between chemists, biologists, engineers, and clinicians is essential to overcome the challenges associated with isolation, characterization, scale-up, and clinical validation [7]. Furthermore, sustainable sourcing and the responsible utilization of natural resources are paramount to ensure the long-term availability of these valuable compounds. By embracing innovation and prioritizing sustainability, we can unlock the full potential of natural products to combat antimicrobial resistance and revolutionize drug delivery, shaping a healthier future for all [8].

## 2. An Overview of the Published Articles

This Special Issue showcases research on natural antimicrobials and enhanced drug delivery systems. The included articles explore the discovery, characterization, and application of novel antimicrobial compounds. They also highlight innovative drug delivery strategies, utilizing natural products to improve bioavailability, target tissues, and reduce side effects. This collection provides a comprehensive overview of the field’s status and future research directions in natural product-based antimicrobial and drug delivery systems.

Cannabidiol (CBD), a compound derived from *Cannabis sativa* L. with anti-inflammatory, analgesic, neuroprotective, and antibacterial properties [9], was incorporated into PURASORB scaffolds. PURASORB 5010/CBD scaffolds exhibited antibacterial activity against *S. mutans* UA159, *S. aureus* ATCC25923, and a multidrug-resistant *S. aureus* clinical isolate (MDRSA CI-M) for up to 17 days, surpassing the antibacterial effectiveness of PURASORB 7510/CBD. Adding PEG400 enhanced the antibacterial activity of PURASORB 7510/CBD but not PURASORB 5010/CBD. In addition, the daily CBD release from these scaffolds (1.12–9.43 µg/mL), below the cytotoxic threshold of 25 µg/mL, effectively reduced LPS-induced IL-6 secretion in RAW 264.7 macrophages without exhibiting cytotoxicity towards RAW 264.7 macrophages or Vero epithelial cells. Therefore, incorporating CBD into biodegradable PURASORB 5010 provides a method for creating sustained-release devices with combined antibacterial and anti-inflammatory benefits (Contribution 1).

Researchers also investigated plant terpenes as natural antimicrobials to overcome bacterial resistance. Terpenes protect against stress and show therapeutic promise, including antimicrobial activity, resistance modulation, anti-inflammation, and immune modulation, exemplified by estragole (ES). Poor solubility limits use, but beta-cyclodextrin (β-CD) encapsulation improves stability, bioavailability, and solubility [10]. The ES/β-CD complex lacked direct antibacterial activity; it synergistically enhanced norfloxacin’s efficacy against the 1199B (NorA) strain, suggesting its potential as an antibiotic potentiator rather than a direct efflux pump inhibitor. Molecular docking simulations indicated a high affinity for ES/β-CD complex formation. Furthermore, pharmacokinetic predictions suggest moderate lipophilicity, high cellular permeability, and low toxicity, supporting its potential as a pharmacologically promising oral adjuvant. These findings indicate that the ES/β-CD complex could be a relevant adjuvant in antibiotic therapy to combat multidrug-resistant bacteria, although in vivo studies are needed to confirm this effect (Contribution 2).

The rise in antimicrobial resistance necessitates exploring alternative agents like essential oils (EOs), which are secondary metabolites with promising antimicrobial potential. EOs can reduce antibiotic dosages and adverse effects when used in conjunction, potentially overcoming resistance through novel synergistic mechanisms. Pinus species are traditionally used and exhibit promising antimicrobial properties; however, the biological activities of essential oils from Bosnian and Herzegovinian Pinus species remain largely unexplored [11]. The chemical analysis of the EOs Pinus species from Bosnia and Herzegovina revealed high concentrations of α-pinene, (*E*)-caryophyllene, limonene, germacrene D, myrcene, and δ-3-carene. *P. sylvestris* EO showed strong efficacy against *S. aureus* and *E. faecalis*, while *P. nigra* EO inhibited *E. coli* at 100 μg/mL. *P. halepensis* EO exhibited the strongest activity against *E. faecalis*, and *P. halepensis* EOs were highly active against *C. albicans*. Synergistic interactions between selected EOs and gentamicin were observed against *S. aureus* and *K. pneumoniae*. *P. sylvestris* and *P. halepensis* EOs demonstrated the greatest antimicrobial activity, and in conjunction with gentamicin, these EOs, along with *P. nigra* EO, showed synergistic potential, supporting their use either alone or with antibiotics and aligning with ethnopharmacological applications (Contribution 3).

Natural products such as brown seaweed are attracting anticancer research due to their bioactive compounds, including sulfated polysaccharides, phlorotannins, carotenoids, and polyphenols, which demonstrate cytotoxic and anti-proliferative activity. These compounds uniquely modulate cancer pathways, such as apoptosis and metastasis, while their antioxidant activity combats oxidative stress. Lung cancer treatment includes chemotherapy, immunotherapy, and nanoparticle drug delivery. Nanoparticles (NPs) improve drug targeting and reduce side effects. In this way, the conjugation between zinc oxide nanoparticles (ZnO NPs), which induce cancer cell oxidative stress, phloroglucinol (PHL), a cytotoxic and anti-proliferative polyphenolic compound extracted from brown algae, and polyethylene glycol (PEG), which reduces immune system recognition and enhances the NPs’ ability to evade clearance mechanisms, thereby extending their circulation time in the bloodstream, (ZnO-PEG-PHL NPs) represent a synergistic strategy for cancer therapy [12]. The purified PHL fraction exhibited a high phenolic content (45.65 mg PHL/g), confirmed by spectral analysis. Upon their incorporation into ZnO-PEG-PHL NPs, the size increased from 32.36 nm to 46.68 nm, and the zeta potential shifted from −37.87 mV to −26.82 mV. The ZnO-PEG-PHL NPs displayed superior antioxidant activity in all assays and demonstrated enhanced cytotoxicity (IC_50_ = 40 µg/mL) compared to ZnO NPs (60 µg/mL) and PHL (70 µg/mL). Apoptotic studies indicated significant cell cycle arrest and apoptosis induction. These results suggest that ZnO-PEG-PHL NPs are promising candidates for cancer therapy and antioxidant applications due to their enhanced antioxidant and anticancer properties (Contribution 4).

*Pistacia lentiscus*, a drought-resistant Mediterranean shrub, is traditionally used as an antioxidant as well as being hepatoprotective, anticancer, and antimicrobial in nature. Lentisk oil (LO), extracted from *P. lentiscus* berries, promotes skin lesion repair, inhibits lipid oxidation, and prevents antioxidant enzyme depletion. LO, traditionally used topically for burns, wounds, and sores, also exhibits antimicrobial activity, which, combined with oil-based nanocarriers, can improve topical treatment effectiveness against multidrug-resistant *S. aureus* [13]. Levofloxacin (LVX), the levo isomer of ofloxacin and a conformationally restricted analog of third-generation norfloxacin, exhibits enhanced potency and reduced toxicity compared to its dextro form. Effective against both Gram-positive and Gram-negative bacteria, LVX also demonstrates significant activity against atypical pathogens like Chlamydia, Mycoplasma, and Legionella. Its lipophilic nature makes it suitable for encapsulation within bioactive oil phases like nanoemulsions (NEs) [14,15]. Our study, employing LVX-LO-loaded Nes as an example of anti-staphylococcal activity, provides a foundation for developing novel antimicrobial therapeutic strategies. The phytochemical characterization of *P. lentiscus* oil (LO) identified bioactive compounds, including palmitic and oleic acids along with stearic and linoleic acids, which were found to be the most abundant fatty acids in LO in addition to flavonoids, monoterpenes (e.g., α-pinene and β-myrcene), and sesquiterpenes (e.g., β-elemene, α-humulene, and α-cubebene), suggesting its potential as an antimicrobial/antibiofilm adjuvant in topical antibacterial formulations. In comparison to the free-form antibiotic, the loaded LVX-LO-NEs exhibited enhanced antimicrobial activity against the sessile forms of *Staphylococcus* spp. strains (Contribution 5).

## 3. Conclusions

In conclusion, this Special Issue underscores the therapeutic potential of natural products delivered via diverse systems to combat microbial infections. This burgeoning field offers promising avenues for addressing the ever-increasing challenges posed by antimicrobial resistance. The included articles highlight innovative approaches, ranging from nano-encapsulation and targeted delivery to specific sites of infection, showcasing the enhanced efficacy and bioavailability achievable through strategic formulation. Furthermore, the diverse array of natural product sources explored underscores the rich reservoir of bioactive compounds awaiting further investigation and clinical translation. As the threat of antibiotic resistance continues to escalate, the development and optimization of natural product-based therapies, facilitated by advanced delivery systems, represents a crucial strategy in the ongoing battle against infectious diseases.
